# Cannabidiol’s Antioxidant Properties in Skin Care Products and Legislative Regulations

**DOI:** 10.3390/plants14223521

**Published:** 2025-11-18

**Authors:** Maria Fafaliou, Apostolos Papadopoulos, Panagoula Pavlou, Athanasia Varvaresou

**Affiliations:** 1 Sector of Aesthetics and Cosmetic Science, Department of Biomedical Sciences, University of West Attica, GR-12243 Egaleo, Greece; magia.fafalios@gmail.com (M.F.); ppavlou@uniwa.gr (P.P.); avarvares@uniwa.gr (A.V.); 2 Laboratory of Chemistry, Biochemistry and Cosmetic Science, Department of Biomedical Sciences, University of West Attica, 28 Agios Spyridonos Street, GR-12243 Egaleo, Greece

**Keywords:** CBD, antioxidant, anti-inflammatory, skincare, cosmetics, regulations

## Abstract

Cannabidiol (CBD) has garnered interest in its potential antioxidant properties in skin care. This review synthesizes the current literature exploring CBD’s role as an antioxidant and anti-inflammatory cosmetic ingredient and its impact on skin health. CBD exhibits antioxidant effects by scavenging free radicals, reducing oxidative stress, and mitigating inflammation, all of which contribute to aging and skin conditions like acne and dermatitis. CBD interacts with the endocannabinoid system and other cellular pathways to bolster antioxidant defenses in skin cells. The search engines were Google Scholar, Scopus, and PubMed. The search was performed using the main keywords “liposomal CBD and keratinocytes”, “antioxidant properties of CBD and keratinocytes”, “anti-inflammatory activity of CBD and keratinocytes”, “CBD and cosmetics”, “CBD and skin care products”, and “CBD and cosmetic products regulation” for the period 2018–2025. The period for the search of the literature was chosen based on the legalization of medical cannabis. Its non-psychoactive nature and favorable safety profile make CBD a compelling candidate for inclusion in skin care formulations seeking natural and effective antioxidant solutions. As consumer demand for botanical-based skin care rises, CBD stands out for its promising therapeutic benefits and potential applications in anti-aging and dermatological treatments. Despite its benefits, using CBD in cosmetics is not without its challenges. The varying legal status of CBD in various jurisdictions is confusing to both the cosmetic industry and consumers. Some jurisdictions allow CBD to be derived from hemp, while others may prohibit its use. The legal and regulatory status of CBD is a constantly changing matter. The purpose of the article is to review the liposomal forms of encapsulated CBD to increase its percutaneous absorption and therefore its antioxidant, anti-inflammatory effect on keratinocytes. Liposomal CBD firstly bypasses the problems of insolubility of free radicals and secondly protects it from various exogenous oxidizing agents, while maintaining its activity. CBD is a valuable antioxidant agent in skin care science, suggesting avenues for further research and product development.

## 1. Introduction

**Figure 1:**
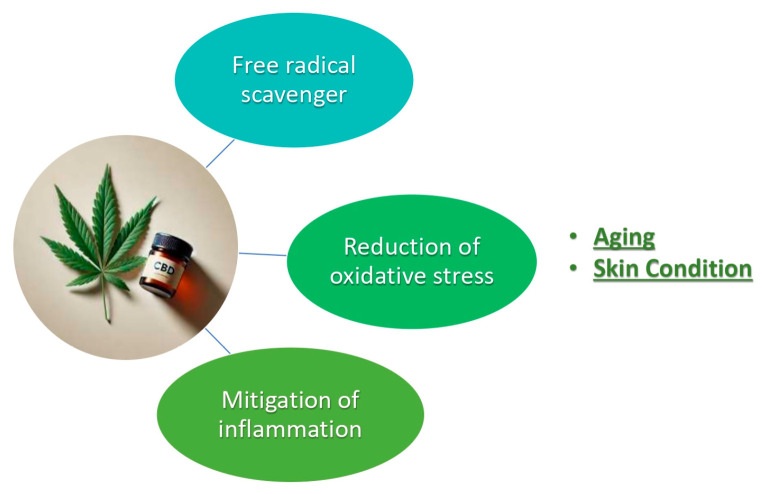
CBD (cannabidiol) and properties by skin protection.

CBD is a prominent non-psychotropic cannabinoid found in the *Cannabis sativa* L. plant. CBD exerts its effects through multiple mechanisms, including the modulation of the endocannabinoid system. It interacts with cannabinoid receptors CB1 and CB2, though with low affinity, and influences various non-cannabinoid receptors such as serotonin 1A receptor 5–HT1A and transient receptor potential (TRP) channels. These interactions result in a broad range of pharmacological effects, including anti-inflammatory, analgesic, anti – anxiety, and neuroprotective properties [1,2,3]. The incorporation of CBD in skin cosmetics is gaining interest due to its potential benefits for the skin (Figure 1). Some of the key properties that make CBD a promising ingredient in skin care formulations are as follows:

- Anti-inflammatory: CBD’s ability to modulate inflammatory responses makes it suitable for treating skin conditions such as acne, eczema, and psoriasis. It causes a reduction in redness, swelling, and irritation.
- Antioxidant: Antioxidants can protect the skin from damage caused by free radicals and UVA and UVB radiation. This helps in slowing down the aging process and preventing the formation of wrinkles and fine lines.
- Sebum Regulation: CBD can influence the production of sebum, helping in maintaining balanced skin moisture and preventing acne breakouts.
- Skin Barrier Function: CBD enhances the skin’s barrier function, which is crucial for maintaining hydration and protecting against environmental stressors. Improved barrier function contributes to overall skin health [4,5].

CBD influences the redox balance by modifying the level and activity of both oxidant and antioxidant CBD and interrupts free radical chain reactions, capturing free radicals or transforming them into less active forms. The free CBD radicals produced can have many resonance structures in which unpaired electrons are mainly localized on the phenolic structure, suggesting CBD antioxidant activity can be attributed to the phenolic groups [6].

The normal redox potential of CBD measured by cyclic voltammetry is about E_0_: +1.21 Volts; therefore, it is easily oxidized by donating an electron [7].

This direct antioxidant action is due to a free radical substitution reaction, in which CBD inactivates environmental free radicals and reactive oxygen species (ROS).

CBD also reduces ROS production by chelating transition metal ions involved in the Fenton reaction to form extremely reactive hydroxyl radicals. CBD, acting similarly to the classic antioxidant butylated hydroxytoluene, prevents dihydrorodamine oxidation in the Fenton reaction [8,9].

The Fenton–Haber–Weiss reaction involves the generation of highly reactive hydroxyl radicals (•OH) through a series of chemical reactions involving iron and hydrogen peroxide (H_2_O_2_). This reaction is significant in biological systems because produced (•OH) can cause oxidative damage to biomolecules, leading to cellular dysfunction and contributing to various diseases, including neurodegenerative disorders and cancers [10].

On the other hand, CBD leads to increased levels of antioxidant enzymes such as superoxide dismutases (SODs), catalases, glutathione peroxidases (GPx), and peroxyredoxins, which are involved in oxygen metabolism and maintaining the balance of oxidative stress [4]. The phenolic ring of olivetol fragment can participate in electrophilic aromatic substitution reaction. In this way, it is possible for CBD to be attacked by nucleophile reagents as the thiol group of a cysteine (Cys). The possibility of the interaction of the olivetol ring with a thiol group of Cys that are parts of the complex of the Keap-1-Nrf2 protein increases the level of proinflammatory factors in the nuclear factor kappa-light-chain-enhancer of activated B cells (NFκB) pathway and partially modifies the cytoprotective activity of nuclear factor erythroid 2-related factor 2 (Nrf2) in keratinocytes [5]. Nrf2 is a transcription factor and regulates cellular defense against free radicals through the expression of genes that transcript antioxidant enzymes involved in oxidative stress. The regulation of various pathological conditions that present redox imbalances and inflammation including cancer, inflammatory diseases, and neurodegenerative diseases is affected by oxidative modifications. Lipid peroxidation is important in such cases as it causes the oxidation of polyunsaturated fatty acids (PUFAs), like linolenic, linoleic, eicosapentaenoic, arachidonic, and docosahexaenoic acids. The reaction of ROS with PUFAs leads to the formation of lipid hydroperoxides and, due to oxidative fragmentation, unsaturated aldehydes like malondialdehyde (MDA), acrolein, and 4-hydroxynonenal (4-HNE). Propagation of oxidation chain reactions, especially regarding docosahexaenoic acid, can cause oxidative cyclization and produce isoprostanes or neuroprostanes. CBD regulates the level of oxidative stress involved in cell signaling pathways, thereby preventing the oxidation of lipids and proteins. In addition, CBD acts by supporting the activity of the biological system since it is a phytocannabinoid. Additionally, because CBD increases levels of anandamide (ANA), which affects cannabinoid signaling and their interaction at cannabinoid receptors, it modulates the activity of the endocannabinoid system [11]. Also, endocannabinoid-activated peroxisome proliferator-activated receptor gamma (PPAR-γ) functions to regulate the expression of antioxidant enzymes such as superoxide dismutase by interacting with their promoter regions [12,13].

An important antioxidant function of CBD is its effect on receptors. The action of CBD depends on its concentration. CBD can lead to activation, antagonism, and inhibition of cannabinoid receptors CB1 and CB2, as well as ionotropic (TRP) and nuclear (PPAR) receptors in a dose–dependent manner.

## 2. Cannabidiol’s Antioxidant and Anti–Inflammatory Properties on Keratinocytes

Skin aging occurs under the influence of environmental free radicals and ROS in conditions of oxidative stress and in inflammatory conditions. Hemp oil has many applications and one of them is its topical application to the skin. It is combined with products containing ceramides, hyaluronic acid, peptides, and niacinamide, with which it works synergistically and deeply nourishes the skin by strengthening the skin’s barrier. Preclinical evidence suggests that topical application of CBD may be effective for certain skin disorders, such as eczema, psoriasis, and inflammatory conditions. CBD suppresses inflammatory reactions due to allergic contact dermatitis in vitro, without cytotoxic effects [14,15].

CBD has been found to modify the redox balance as it changes the level and activity of antioxidants and is also responsible for the transcription of cytoprotective genes, including some antioxidant genes. It was reported that CBD reduced the redox imbalance caused by exposure to UVB/hydrogen peroxide in keratinocytes, estimated by superoxide anion radical generation, total antioxidant status, and consequently lipid peroxidation product level. The protective effect of CBD on viability of keratinocytes, melanocytes, and skin fibroblasts following UV irradiation was also recently shown [9].

Sermet et al. [16] examined the anti-inflammatory effects of CBD and found that it was able to prevent an increase in tumor necrosis factor mRNA expression. It was also able to normalize lipopolysaccharide (LPS)-induced expression of Interleukin 1β (IL-1B) and Interleukin 6 (IL6). These data provide further insight into the key anti-inflammatory effects of CBD. Specifically, the control of sebaceous cell proliferation and lipid production is thought to be linked to ion channel protein transient receptor potential cation channel subfamily V member 4 signaling [16]. Hwang et al. have shown that CBD stimulates the activity of both melanin and tyrosinase, which is induced by CB1 receptors in human epidermal melanocytes [17].

CBD induces expression of Hemooxygenase 1 (HMOX1) and other Nrf2-regulated genes, as shown in in vitro studies. A study conducted on normal human epidermal keratinocytes analyzed expression of several Nrf2 target genes that CBD induced, with the most upregulated being HMOX1 [2]. After topical application of CBD, an increase in the levels of HMOX1 and keratins 16 and 17 (wound repair keratins) in mice epidermis was noticed [2].

CBD was able to balance the oxidative stress response resulting from cell penetration in human keratinocytes exposed to UVB irradiation and hydrogen peroxide. It has also been demonstrated that CBD protects membranes’ integrity against peroxide-induced reduction in polyunsaturated fatty acids [3].

Studies suggest that CBD can activate PPAR-γ. Two-dimensional and three-dimensional fibroblast cells were treated with it, resulting in the activation of PPAR-γ and a decrease in levels of NF-kB [4]. Using HaCaT cells in an in vitro model of allergic contact dermatitis, CBD was found to reduce cytokine production significantly [13].

Glutathione (GSH) acts as an antioxidant in synergy with other low molecular weight compounds, mainly with vitamins such as A, E, and C [18]. CBD behaves as a negative allosteric modulator for the CB1 and CB2 receptors, as it antagonizes other agonists [19,20]. CBD, through the inhibition of fatty acid amide hydrolase, stimulates the release of endocannabinoid ANA, simultaneously activating a number of other receptors [21]. CBD-stimulated ROS generation in mitochondria was accompanied by apoptosis in monocytes [22]. MDA consideration can be reduced with the use of CBD, whose inflammatory properties through repeating doses have been found to augment GPx and reductase activity in studies with a control group [23]. CBD treatment of human keratinocytes irradiated with UVB has shown similar results vis-à-vis GSH levels and GPx activity. Cannabidiol hydroxyquinone, a reactive metabolite of CBD, can inhibit the activity of GSH and cytochrome P450 3A11 by forming adducts with them as it reacts with Cys with covalent bonds. The activity of Indoleamin-2,3-dioxygenase can be decreased by CBD, thus protecting tryptophan from degenerating. The antioxidant potential is highly dependent on the experimental conditions and the method chosen. CBD may have a higher antioxidant potential if studied in an oil-based environment [24]. CBD exhibits good stability, whether or not it has been irradiated with UVA radiation. Ιt induces the UVA (A) and UVB (B) protectivity of non-psychotropic cannabinoids on normal human dermal fibroblasts and human keratinocytes (HaCaT), *n* = 4 [12].

Furthermore, CBD was found to decrease β-amyloid formation in neurons through reduction in the concentration of transition metal ions. Research shows that it has increased the mRNA level of SOD and the enzymatic activity of Cu and Zn- and Mn-SOD, which were responsible for metabolizing the superoxide radicals in a mouse model of diabetic cardiomyopathy type I and in human cardiomyocytes treated with 3-nitropropionic acid or streptozotocin [17]. Studies of the antioxidant and cytoprotective properties of CBD are summarized in Table 1.

## 3. Delivery Systems and CBD

CBD has a very low water solubility (12.6 mg/L), since it is a lipophilic molecule with a logP value of 6.3, where logP is the logarithm of a drug’s partition coefficient between n-octanol and water. Therefore, the dermal permeation from cosmetics using different delivery systems has been examined [25].

Nano formulations seem to enhance CBD solubility, encapsulation efficiency, and stability and sustain CBD release in comparison with other delivery systems. The encapsulation of CBD in liposomes allows for diffusion into deeper layers and its targeted release into cells. In vitro diffusion studies using human tissue have demonstrated the ability of CBD to be transported to the deeper layers of the skin [15].

Preclinical and clinical studies have contributed to the understanding of the potential of CBD to treat many diseases, including those related to oxidative stress. CBD was studied on the one hand in liposomes; on the other, it was studied in a different encapsulation system as well as in a non-encapsulated form. According to research carried out in 2020, microcapsules [encapsulation systems] were prepared using the melt-print technique for polymers. CBD was encapsulated in the cage system and injected subcutaneously into mice. It was observed that the CBD was gradually released over the next two weeks, which was also seen by its concentration in the blood. One week after administration, the frequency of seizures decreased by 40% and the survival rate increased by 50% [26]. In 2003, Lodzki et al. reported the successful transdermal delivery of CBD using ethosomal carriers in a murine model [27].

Another clinical safety report was performed on 10 volunteers who received a liposome-encapsulated CBD preparation. A total of 340 blood tests were performed, of which 339 showed stable values or changed towards ideal values. Also, initially there were five participants with elevated blood glucose value on the initial day (day 0), who had normal glucose levels at the end of the study on the 30th day (day 30) [28].

The efficacy of topical application of CBD (1–10%) in gel form, specifically for reducing symptoms associated with arthritis inflammation, was investigated and found to be well absorbed. The amount absorbed expressed as a concentration showed a linear relationship with the applied dose. The topical application of CBD has therapeutic potential to relieve pain and inflammation associated with arthritis pain without apparent side effects [29].

Another research paper refers to normal human keratinocytes in which CBD acted by causing the expression of several target genes for Nrf2, with the enzyme-encoding gene HMOX1, which has antioxidant and anti-inflammatory properties, being the gene regulated most by CBD [30].

In another scientific study, an in vitro study was conducted to treat glioblastoma. Both liposomes loaded with CBD and empty liposomes were examined. It was observed that the smaller-sized liposomes (20 nm) resulted in a threefold reduction in the IC50 value compared to the larger-sized liposomes (50 nm) [31].

A study conducted with the participation of 15 volunteers measured the percentages of CBD in the blood after participants took, per os, non-encapsulated CBD and a formulation with CBD encapsulated in liposomes. A total of 100% of participants receiving the liposome formulation had CBD in their blood, while for participants receiving the non-encapsulated CBD formulation, only 40% had CBD levels in their blood [32].

The potential use of CBD in solution and encapsulated in polymeric microparticles when combined with paclitaxel (PTX) and doxorubicin (DOX) in the treatment of breast cancer was examined. The administration of CBD solution enhanced the effectiveness of PTX and DOX in both types of breast cancer cells tested. The parallel administration of CBD solution and PTX or DOX showed a synergistic effect. The designed formulation of CBD in microparticles was effective, with prolonged antiproliferative activity for at least 10 days when combined with PTX or DOX. Also, the formulation showed a significant increase in the antiproliferative activity of PTX and DOX [33]. According to an in vitro study with human keratinocytes, it was reported that CBD was able to penetrate them and restore the balance of oxidative stress that had been stimulated by UVB radiation. CBD was found to significantly enhance the activity of antioxidant enzymes such as SOD and thioredoxin reductase in UV—irradiated keratinocytes [9].

It was also observed that CBD had an antioxidant and protective effect against the degradation of the cell membrane caused by the action of free radicals forming peroxides of PUFAs. This is supported by a study, in which the HPLC/MS technique was used to measure the transport of CBD into keratinocytes. In a first step, the keratinocytes were irradiated with UVB radiation to induce oxidative stress and the consequent uncontrolled production of free radicals. The change in the concentrations of peroxidized lipids/intact lipids before and after the administration of liposomal CBD was then observed by HPLC/MS. The administration of liposomal CBD led to a decrease in the ratio of peroxidized lipids/intact lipids, t [34].

Another study was conducted to evaluate the efficacy of a dermal membrane structure liposome cream bearing primarily CBD for the treatment of pruritus in hemodialysis patients. After three weeks of treatment, pruritus was completely eliminated in eight patients (38.1%). The three-week treatment period resulted in a complete reduction in dryness in 17 patients (81%) [35]. Administration of free CBD to a culture of human sebocytes appeared to inhibit the adipogenic actions of various compounds, including arachidonic acid and a combination of linoleic acid and testosterone, suppressing sebocyte proliferation. These findings suggest that due to its combined lipostatic, antiproliferative, and anti-inflammatory effects, CBD can be useful for the management of acne vulgaris. The results of the above studies regarding the antioxidant and anti-inflammatory activity of liposomal and non-encapsulated CBD firstly indicate that a quantity of CBD is released into the cell cytoplasm and is then involved in oxidative stress control mechanisms by suppressing the overproduction of free reactive forms of oxygen in oxidative stress. Secondly, the release of CBD is observed in the plasma membrane of keratinocytes, which inactivates free radicals with a direct mechanism of action and provides protection against the peroxidation of phospholipids [36].

McCormick et al. [37] produced a nanoparticle-encapsulated CBD cream that protected against nuclear and mitochondrial DNA mutations associated with UV-A-induced skin aging. They showed that samples treated with this form of CBD exhibited less severe UV-induced epidermal hyperplasia, a decrease in UV-A-induced epidermal hyperplasia, an increase in the premutagenic marker 8-Oxoguanine glycosylase, and a decrease in two significant UV-A-induced mtDNA deletions linked to skin photoaging. The studied CBD cream demonstrated that this medication delivery method was successful in applying the target active component topically. As an antioxidant, CBD influences the redox system directly and interacts with related molecular targets to produce indirect antioxidant effects [37].

## 4. Mechanism of CBD’s Transport from Liposomes to the Cell Membrane

Liposomes consist of a bilayer of phospholipids. Thus, when they come into contact with the skin, they penetrate the stratum corneum, as the cell membrane follows the model of the fluid mosaic, which enables the phospholipids to move on its surface and the liposome to integrate into it and be part of it. So, CBD that was trapped inside the liposome bilayer is now inside the cell membrane bilayer [38,39].

A necessary factor for the fusion of the cell membrane with the liposome are membrane proteins. The proteins involved are the Soluble NSF Attachment Receptor proteins (SNARE) and N-ethylmaleimide Sensitive Fusion protein (NSF), the RAB GTPase protein, and the RAB Effector protein. SNARE proteins are divided into T-SNARE and V-SNARE proteins. Ts are on the target membrane and Vs are on the carrier membrane. The liposome can bear a RAB GTPase protein, which recognizes the target membrane, while the RAB Effector protein is found in the cell membrane and is a connecting protein [40].

The process of membrane fusion with the liposome has three stages: the connecting stage, the docking stage, and the fusion stage. During the first stage, the RAB GTPase protein of the liposome recognizes the RAB Effector protein of the membrane, and they bind to each other. During the docking stage, the SNARE protein Ts of the membrane and Vs of the liposome are, respectively, recognized and connected, resulting in the trans SNARE complex. Then the GTPase of the RAB protein is hydrolyzed to guanosine diphosphate and thus a soluble RAB GDP molecule is obtained, which is disconnected from the two molecules, and thus the connection of the two membranes is made. Finally SNARE proteins form a cis trans SNARE complex, which is hydrolyzed by NSF and dissociated. This is a process that cells use to transport various substances, proteins, etc., to specific cells or cell organelles. The possibility of isolation and the following incorporation of necessary SNARE proteins into liposomes increase the chances of their fusion with cell membranes. According to in vitro studies, fusion is possible only in the presence of complex SNARE proteins, i.e., Vs and Ts, without the presence of RAB proteins [41].

The most common is the synaptic SNARE complex consisting of synaptobrevin/Vamp in the vesicle and syntaxin 1 and SNAP-25 in the plasma membrane. SNARE proteins bind in a cluster of four parallel helical bundles, with SNAP-25 providing two and the other two proteins providing one sequence each. Assembly of the cluster proceeds by a zipper-like process capable of providing energy for fusion [42]. Additionally, a change in lipid composition can lead to pH-sensitive liposomes, causing increased fusionogenic effects in regions of low pH, such as endosomes. Lipids that can form no bilayer phases, such as dioleoylphosphatidylethanolamine, lead to destabilization of the bilayer and promote fusion [43].

One targeting mechanism exhibited by liposomes is active internal targeting, that is, the cell membrane receptor mechanism, which allows the receptors a specialized interaction between liposomes and cells. Liposomes carry targeting ligands which bind to the corresponding cell receptors, among which are folic acid, sugars, i.e., galactose and lectins, modified albumin, peptides, and antibodies.

## 5. Radical Chain Mechanism by the Neutralization of Free Radicals in Keratinocytes by CBD

S. Atalay et al. [3] reported that the radical chain reaction of CBD with ROS under conditions of oxidative stress, i.e., by irradiation of UVB in combination with H_2_O_2_, lead to interruption of the chain reactions. The action of CBD is the neutralization of a superoxide free radical (•HOO) under the conditions of oxidative stress.

**Figure 2:**
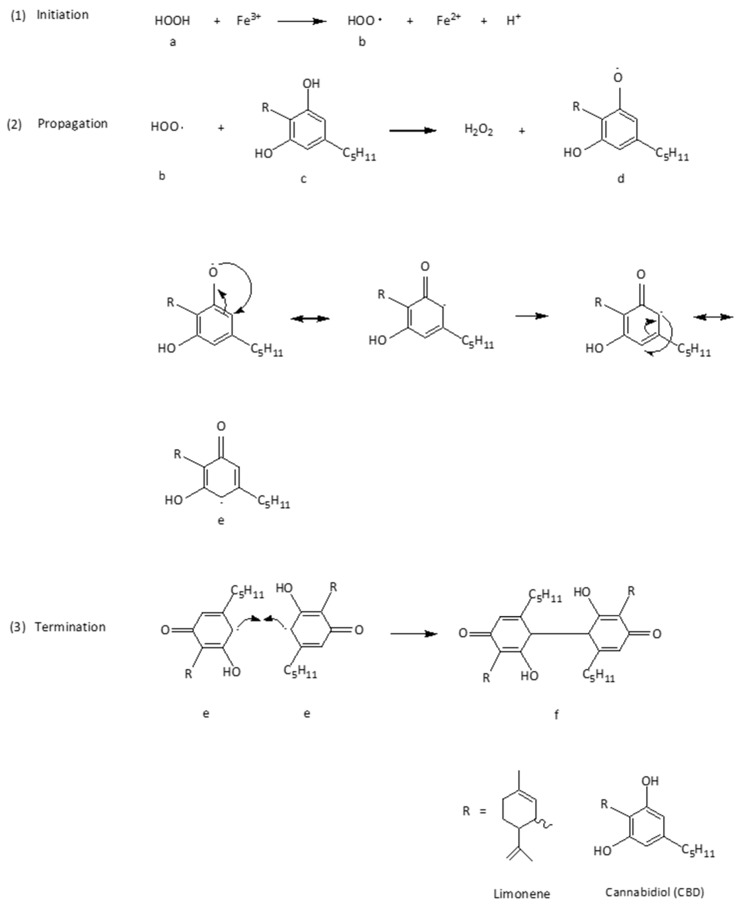
Radical chain mechanism of CBD. (a: hydrogen peroxide, b: superoxide free radical, c: CBD, d, e: CBD-free radical, f: dimerized p-semiquinone).

A mechanism has been proposed for the action of CBD in scavenging free radicals within the membrane bilayer of keratinocytes. According to this mechanism (Reactions 1: initiation step) (Figure 2), H_2_O_2_ undergoes oxidation via the Fenton reaction, reducing Fe^3+^ to Fe^2+^. H_2_O_2_ is thus converted into a •HOO (b). In the propagation step, a proton substituent from the phenolic ring [olivetol moiety] of CBD (c) is homolytically split and reacts with •HOO (b), which is thereby neutralized and converted back to H_2_O_2_ (c), which can be neutralized by physical antioxidants of the keratinocytes. Simultaneously, a CBD-derived free radical (semiquinone) is formed (d, e), wherein the unpaired electron is delocalized over the aromatic ring due to resonance [6]. The termination of the chain reaction could be stopped by the combination of two low-energy CBD radicals (e) building the dimerized p-semiquinone (f).

**Figure 3:**
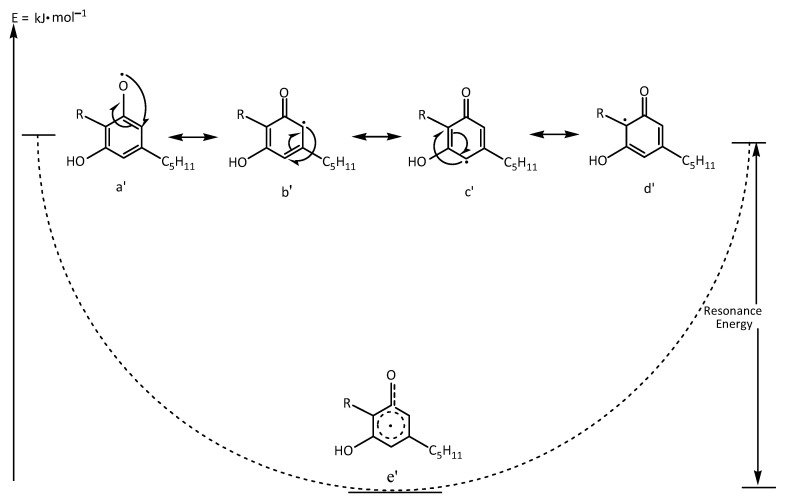
Resonance structures of the CBD showing the electron delocalistion of the instable radical resonance hybrid a′, b′, c′, d′ and the energy lowest stable structure of e′.

The stability of the resulting free radical is enhanced by the extent of resonance delocalization. The more resonance hybrid structures a molecule can exhibit for delocalizing an unpaired electron, the more stable the radical becomes. In the case of CBD, the unpaired electron localized on the olivetol ring (d) can be delocalized over four resonance structures. This results in a decrease in the energy of the free radical and, consequently, in its reactivity (d). The stability of the resonance structures (a′, b′, c′, d′) (Figure 3) is further influenced by the substituent group R (ligand) in the ortho position, which is in hyperconjugation with the carbon of the phenolic ring bearing the delocalized unpaired electron in the resonance hybrid (d′). Overall, resonance is described by the hybrid structure (e′), which has lower energy relative to the individual resonance forms (a′, b′, c′, d′, e′) (Figure 3).

**Figure 4:**
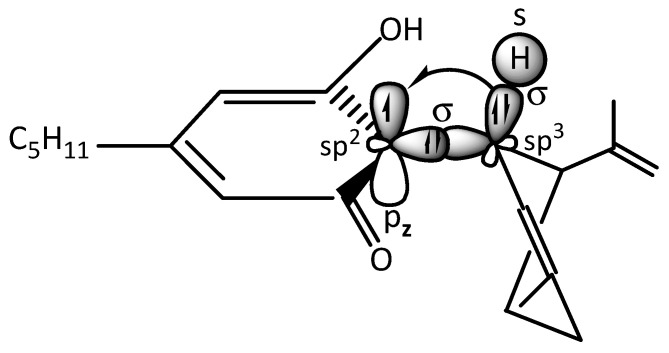
Hyperconjugation between σ_(sp_^3^_-s)_ electron of carbon atom of limonene and p_z_ electron of sp^2^ carbon atom of olivetol ring.

**Figure 5:**
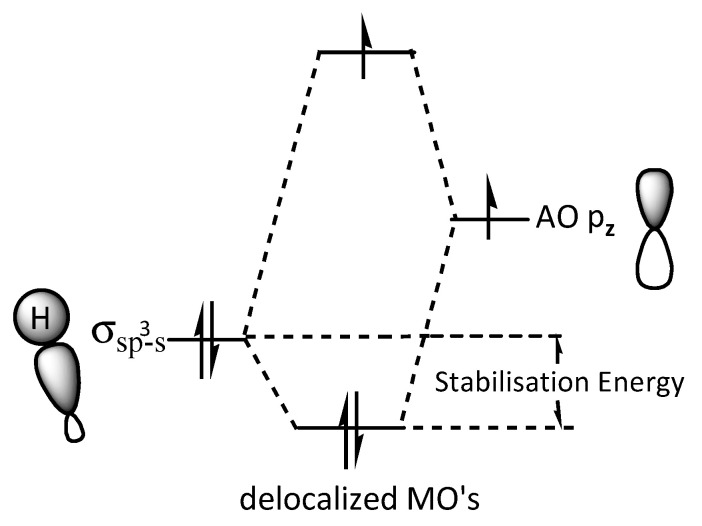
MO (molecular orbital) model of radical hyperconjugation. 3e-stabilizing interaction between a filled donor orbital σ_(sp3-s)_ and a half filled acceptor p_z_ atom orbital, leading to low stabilisation energy.

In the CBD radical, an sp^3^ hybridized orbital of a carbon atom in the limonene ring overlaps frontally with an sp^2^ hybridized orbital of a carbon atom in the olivetol moiety, forming a σ_(sp_^3^_-sp_^2^_)_ bond. The unpaired electron in the sp^2^ carbon resides in a p_z_ orbital. Electron donation from a σ_(sp_^3^_-s)_ C–H bond into the p_z_ orbital increases its electron density and stabilizes the free electron p_z_ orbital (Figure 4). According to molecular orbital (MO) theory, the σ_(sp_^3^_-s)_ C–H bond overlaps laterally with the p_z_ atomic orbital of the carbon bearing the unpaired electron, leading to the formation of a bonding (MO) (occupied by the C–H electron pair) and a non-bonding MO (occupied by the unpaired electron) (Figure 5). The net effect of this hyperconjugation is a positive energy balance favoring the stabilization of the free radical [44,45].

Additionally, the phenolic group at the meta position to the carbonyl group exerts a positive mesomeric effect (+M effect) (Figure 4), increasing the electron density of the aromatic system and further contributing to the stabilization of the unpaired electron on the phenolic ring.

## 6. CBD in Cosmetic Industry, Skin Care Products, and Legislative Regulations

The global CBD skin care market size was valued at USD 1.89 billion in 2023. It is estimated to reach USD 12.62 billion by 2032, growing at a Compound Annual Growth Rate of 23.5% during the forecast period 2024–2032. The market demand for CBD skin care products is stimulated by factors such as the products’ efficacy in treating skin–related disorders. Producers are marketing CBDinfused skin care products as plant-origin cosmetics on the international market after realizing their distinctive qualities. Leading market players are collaborating to introduce or promote new products [46].

The market for CBD skin care products has been divided according to product type, such as oils, cleansers, masks and serums, lotions and creams, and sunscreens, with oils being the predominant form. The percentage of oil forms is growing because CBD skin care oils are used to treat age-related skin issues and acne. The anti-inflammatory qualities of CBD oils help wound healing without causing irritation [47,48,49]. The external signs of aging are lessened by the antioxidant qualities of CBD oils. They include two fatty acids (omega 3 and omega 6), which help the skin maintain hydration and prevent water loss, a particularly beneficial property for older adults or those more conscious of signs of aging [50].

The use of CBD in cosmetics is not without difficulties, despite its advantages. Both consumers and manufacturers are confused by the highly regulated cosmetics sector and the inconsistent legal status of CBD across different regions. Hemp-derived CBD is permitted in some countries, while its usage may be restricted in others. CBD’s current legal and regulatory status is a continuously evolving issue. CBD skin care products are strongly regulated by authorities on account of their tetrahydrocannabinol (THC) content. A THC level over 0.3% indicates that the CBD is considered marijuana. Internationally, there is no real regulatory harmonization and it is not easy to know which countries accept the presence of CBD in cosmetic products. For some it is strictly forbidden, as is the case for China and Brazil, for example.

The Food and Drug Administration (FDA) in the United States declared on 25 July 2019 that hemp plants are officially approved for use in products. Since CBD is accessible to consumers and contains 0.3% THC or less, it is legally available in the United States. To date, the FDA has approved one cannabis-derived drug product, Epidiolex^®^ (CBD), and three synthetic cannabis-related drugs, Marinol^®^ (dronabinol), Syndros^®^ (dronabinol), and Cesamet^®^ (nabilone). These drugs are only available with a prescription from a licensed healthcare provider [51]. Epidiolex^®^ is included in Schedule V of the Controlled Substance Act list of illegal substances, while CBD itself is listed in Schedule I, along with THC and marijuana [51]. Pharmaceutical formulations with restricted concentrations of specific narcotics are included in the corresponding Schedule V [51].

Cosmetics with CBD as an active ingredient have been registered by Cornbread Hemp in the Louisville, KY, USA.

(1) CBD Lotion Skin Formula 750mg (CBD: 1.616%, THF: 0.611%, Total Cannabinoids: 1.529%) [52].
(2) Peppermint and Arnica CBD Balm 750 mg (CBD: 1.198%, THC: 0.052%, Total Cannabinoids: 1.323%) [53].
(3) CBD Lotion + Menthol 750 mg (CBD: 1.486%, THF: 0.045%, Total Cannabinoids: 1.571%) [54].

Since 2018, the FDA has established a THC concentration limit of ≤0.3% for *Cannabis sativa* L. products, including cosmetics [55]. It is noted that commercially available cosmetics containing CBD also contain CBD derivatives at the permitted concentration, as is indicated in the certificates of analysis of these products.

### 6.1. Legislation of CBD in Different Continents and Countries

#### 6.1.1. North America

The US operates under a dual legal system where federal and state laws often conflict. Since 2018, CBD derived from *Cannabis sativa* L. has been legalized at the federal level, provided it contains less than 0.3% THC. However, individual states have the power to impose their own regulations, leading to a patchwork of laws. Some states have embraced CBD, while others maintain strict restrictions. Canada has adopted a more nuanced approach to cannabis legislation, which regulates both cannabis and CBD products. Under this law, all CBD products must come from licensed producers and meet strict safety and labeling requirements. This regulatory framework aims to ensure consumer protection and product quality across the market. Mexico is in the process of developing its regulatory framework for CBD. Recent legislative efforts have focused on establishing guidelines for the cultivation, production, and sale of CBD products. As these regulations take shape, Mexico is poised to become a major player in the North American CBD market.

#### 6.1.2. Latin America

In Colombia, a 2017 decree regulated the cultivation and manufacture of cannabis and its derivatives for medical and scientific purposes. Cannabis cultivation is legal under a license issued by the government and granted by the Ministry of Justice. Those who wish to produce CBD and other cannabinoids must obtain a license from the Ministry of Health. Personal cultivation and consumption of cannabis in Colombia is decriminalized, but its commercial sale is prohibited. CBD can be obtained as a prescription drug or without a prescription as a dietary supplement or cosmetic. In Ecuador, as of February 2021, processed foods and dietary supplements may include all parts of cannabis or its non-psychoactive derivatives as an ingredient, with a THC concentration of less than 0.3% in the final product. In non-psychoactive cannabis, the THC content must be less than 1%, including cannabinoids, isomers, acids, terpenes, salts, and isomer salts used or intended to be used as a raw material for the production of the final product. In Brazil, CBD was legalized for medical use in 2015. However, *Cannabis sativa* L. remains listed as a plant that is prohibited from cultivation. In December 2019, the Brazilian Health Regulatory Authority (Agência Nacional de Vigilância Sanitária) authorized the production and importation of cannabis products. The Resolution is valid for five years. In this Resolution, “Cannabis Product” refers to any product manufactured for medical use and containing, as active ingredients only, plant derivatives or phytopharmaceutical products. Local manufacturers must import the active ingredient in the form of a plant derivative, phytopharmaceutical, bulk, or industrialized product. The law only allows oral or nasal administration, and CBD can only be consumed with a medical prescription. In October 2021, Anvisa published a list of cannabis products that will be automatically approved for individual import by Brazilian patients.

Similarly, in Argentina, Chile, Paraguay, and Peru, CBD is only legal for medical use. In Bolivia, Guyana, Suriname, and Venezuela, CBD is still illegal [56].

In Asia, the legality of CBD varies from country to country. In Singapore and Indonesia, its use is prohibited, while in Japan and Hong Kong, its distribution is permitted. The main criterion for the distribution of CBD in cosmetics is the THC content.

China is one of the largest producers of cannabis. CBD in cosmetics is legal under its legislation. There are three derivative ingredients that are permitted: the fruit, the seed oil, and the leaf extract of *Cannabis sativa* L. In all products, the THC limit must be ≤0.3%. In Hong Kong, cannabis cultivation is illegal, unlike in mainland China. However, CBD in cosmetics is legal as long as its THC content is zero. In Japan, CBD is legal as long as it does not contain any THC. Cannabis legislation defines cannabis as the plant *Cannabis sativa* L. and its products. The definition does not include mature cannabis stalks and their products (except resin), as well as cannabis seeds and their products. Cannabis cultivation is legal in Japan under certain conditions [57].

In Europe, the cultivation of *Cannabis sativa* L. varieties is permitted, provided that they are listed in the EU’s “Common Catalogue of Varieties of Agricultural Plant Species” and the THC content does not exceed 0.2%. Each European country has its own unique attitudes and regulations regarding CBD. However, national laws may have slight differences in THC content. The United Kingdom has established a framework that allows the sale of CBD products, including cosmetics, provided they contain less than 0.2% THC. The Food Standards Agency oversees this regulation, requiring companies to submit safety assessments for their products. Germany allows the sale of CBD products, provided they are derived from hemp and contain less than 0.2% THC. In France, CBD is legal, but products must be derived from hemp varieties with low THC content. Italy has embraced CBD, allowing it to be sold under certain conditions. CBD products must be derived from hemp and contain less than 0.6% THC. In Spain, the handling of CBD is somewhat unclear. While CBD is not classified as a narcotic, the sale of CBD products is often restricted to online platforms. Local regulations can vary significantly, creating a patchwork of enforcement across regions [58].

Within the parameters of Regulation (EC) 1223/2009, the European Commission launched a consultation in June 2023 regarding the safety of CBD used in cosmetic products. The following forms of request for a safety assessment on CBD are being made by the European Commission to the EU Scientific Committee on Consumer Safety, in its pure form when used in cosmetic products or as an extract that may contain contaminants of other cannabinoids, such as THC in trace amounts. The Commission is requesting information on the safety of CBD as part of this evaluation [59]. The World Health Organization Expert Committee on Drug Dependence proposed changes to the Single Convention of 1961. Specifically, they suggested that a footnote be added stating, “All preparations containing predominantly CBD and no more than 0.2% THC would not be under international control.”

Cannabis use and distribution are illegal in Hungary. Only seeds that are metallically sealed may be utilized, and cultivation is only allowed in designated regions. After flowering, industrial hemp with concentrations of THC below 0.3% is suitable for both industrial and medical uses in Austria. CBD oil is legal, and some formulations are utilized, including Sativex^®^, which has 2.7 mg of CBD and 2.5 mg of THC and can be taken with a prescription, and Dronabinol^®^, which also has 2.5 mg of THC.

According to data from the European Monitoring Centre for Drugs and Drug Addiction, hemp is not illegal in the Czech Republic; nonetheless, authorities have allowed a maximum of 0.3% THC to be used in the production of fibers and seeds. THC and five other isomers are listed in the class of drugs in Schedule I of the official Gazette of Romania. Prohibited plants and drugs without a medicinal effect are included in this table, which was created in 2005 and updated in 2018. CBD is not covered in Schedule ΙΙ (official Gazette of Romania) which also contains narcotic plants and drugs with potential medical uses but severe regulations, including the cannabis plant, cannabis resins, tinctures, extracts, and dronabinol [60].

As of right now, the National Drug Agency in Romania has not authorized any medicinal formulations that contain CBD. Many CBD-containing products state that, as CBD is not on the list of forbidden substances, it is regarded as legal.

Another thing to keep in mind is that hemp plants typically contain less than 0.3% THC (depending on the country), whereas hemp seeds only contain trace amounts of THC and CBD. The typical range for CBD concentration is 12–18% [61]. According to a study that measured both THC and CBD levels, it was found that while the content of THC in resin stayed constant in the Netherlands, it rose in the UK, Italy, and France. Additionally, we may point out that resins typically contain higher levels of THC while having little to no CBD. Since the THC content in resins has been rising while the CBD content has either remained constant or drastically dropped, we can suppose that the THC:CBD ratio has increased [62]. Although the limit of the concentration of THC has been strictly determined, the concentration of CBD has not been defined. A concentration of up to 1% is considered acceptable, but there is no legal regulation.

Although no systematic reviews are available in the literature regarding topical CBD application as a cosmetic ingredient, it seems that CBD in skin care formulations has a rather low toxicity profile. Adverse effects regarding dermatological application of CBD have rarely been reported and are mild, i.e., itching and perilesional redness [63].

Many products bear therapeutic claims to seemingly convey they are safe/healthy. In a survey conducted by Dowd et al. [64] of a total quantity of 105 products, 28% of them made therapeutic claims, 14% cosmetic claims, and only 47% remarked that they were not FDA-approved. These results underline the necessity of appropriate regulatory monitoring of products containing cannabis to guarantee product quality and claim substantiation and discourage the use of false or deceptive health claims in the market [64].

#### 6.1.3. Stability, pH

CBD has shown moderate permeability in Parallel Artificial Membrane Permeability Assays and Franz cell diffusion assays. It is stable in the presence of surfactants, including Tween 20, under slightly acidic conditions (pH = 5 and 6). W/O emulsions are considered favorable systems for the incorporation of lipophilic CBD. However, studies have shown that hydrophilic gels incorporating CBD via specific delivery systems are effective regarding CBD permeability and its retention in the skin [65].

#### 6.1.4. Quality Control of CBD in Cosmetics

Regarding the quality control of CBD in cosmetics, liquid chromatography with tandem mass spectrometry (LC-MS/MS) and HPLC-UV-MS/MS have been applied [66,67].

## 7. Conclusions

CBD interrupts free radical chain reactions, capturing or transforming free radicals into less active forms. These free radicals are characterized by many resonance structures where unpaired electrons are mainly found on the phenolic structure; this suggests that the hydroxyl groups of the phenol ring are mainly responsible for CBD antioxidant activity. It would be interesting to investigate the dermal permeation of CBD cosmetics in the presence of other active ingredients with antioxidant activity. The possibility of the synergism between CBD and other well-known antioxidants with the same mechanism of action., i.e., radical chain reaction, could also be examined.

Superoxide radical generation, which is mainly produced by xanthine oxidase and NADPH oxidase (NOX1 and NOX4), is reduced by CBD. CBD chelates transition metal ions involved in the Fenton reaction to form extremely reactive hydroxyl radicals, thus reducing ROS production. Studies have shown that CBD can prevent dihydrorodamine oxidation in the Fenton reaction. The physical properties and function of cell membranes are directly affected by the peroxidation products generated in them. The unsaturated aldehydes formed due to their electrophilic character and structure [carbonyl groups and carbon–carbon double bonds] are chemically reactive molecules that readily form adducts with the most nucleophilic components of the cell, including lipid DNA, GSH, and proteins. One such example is 4-HNE, which has been identified as an activator of the cytoprotective transcription factor Nrf2, a proinflammatory factor acting through the NFκB pathway and an inhibitor of antioxidant enzymes. These reactions result in a decrease in the level of reactive lipid peroxidation products and an increase in the production of protein adducts that promote cell signaling disorders, thereby causing modifications in their metabolism that can lead to dysfunction and apoptosis of cells. Liposomal encapsulation of CBD significantly improves its stability, absorption, and bioavailability, ensuring its transport to deeper skin layers and enhancing its efficacy. This delivery system has been shown to release CBD gradually, ensuring sustained action and improved clinical outcomes.

Preclinical and early clinical studies suggest that CBD holds significant potential as an active ingredient in dermatological and cosmetic formulations. Furthermore, it contributes to skin barrier reinforcement by promoting keratin expression and lipid stability, regulates sebum production through TRPV4 signaling, and supports wound healing and pigmentation balance via modulation of CB1 receptor activity. Additionally, the incorporation of liposomal CBD into skin care formulations represents a promising strategy for maximizing its antioxidant and anti-inflammatory benefits, particularly in dermatological applications. However, current evidence remains limited by the predominance of in vitro and animal models, while human clinical data is still scarce. Variability in CBD formulations, purity, and delivery systems complicates the comparison of results across studies, and dose-dependent effects may influence its safety and efficacy profile. Additionally, regulatory inconsistencies and the absence of long-term safety assessments further restrict the translation of these findings into routine skin care use. Collectively, while CBD shows strong mechanistic potential for managing oxidative stress and inflammation in the skin, further standardized and controlled human studies are necessary to confirm its therapeutic and cosmetic value.

## Figures and Tables

**Table 1:** Summary of studies of the antioxidant and cytoprotective effects of cannabidiol (CBD) in keratinocytes and related skin cell models.

Study/Reference	Model/Cell Type	Study Design and Treatment	CBD Concentration/Dose	Main Findings/Results	Mechanism/Pathways
[9]	Human keratinocytes, melanocytes, fibroblasts	UV- or UVB-induced oxidative stress model; pre- and post-treatment with CBD	1–10 µM (typical range)	CBD reduced ROS generation, restored antioxidant balance, decreased lipid peroxidation, and improved cell viability	Antioxidant defense enhancement, protection of membrane integrity
[2]	Normal human epidermal keratinocytes (NHEK); in vivo mouse epidermis	In vitro CBD treatment and topical application in mice	1–5 µM (cells); 1–2 mg/cm^2^ (topical)	Upregulation of Nrf2 target genes (HMOX1, NQO1, GCLC); increased keratins 16/17 expression (wound repair)	Activation of Nrf2–ARE pathway; induction of cytoprotective enzymes
[3]	Human keratinocytes exposed to UVB and H_2_O_2_	CBD pre-treatment before oxidative challenge	4–10 µM	CBD maintained redox balance, protected polyunsaturated fatty acids from peroxidation	Membrane stabilization; antioxidant modulation
[17]	Sebocytes, keratinocytes (inflammatory model)	LPS-induced inflammation; CBD co-incubation	5–10 µM	Inhibited TNF-α, IL-1β, and IL-6 expression; normalized lipid production	Anti-inflammatory via TRPV4 and NF-κB pathways
[12]	HaCaT keratinocytes (allergic contact dermatitis model)	In vitro cytokine assay with CBD treatment	1–10 µM	Reduced cytokine release and inflammatory markers	Downregulation of NF-κB signaling
[23]	Human keratinocytes (UVB-exposed)	CBD repeated doses vs. control	2.5–10 µM	Increased GSH levels and GPx/reductase activity; decreased MDA	Enhanced enzymatic antioxidant defense
[24]	In vitro (various models, oil-based and aqueous)	Comparative antioxidant capacity assays	Variable	Antioxidant potential higher in oil-based systems; CBD stable under UVA/UVB	Chemical stability; Matrix-dependent antioxidant activity

## Data Availability

All data generated during this study are included in this published article.

## Abbreviations

The following abbreviations are used in this manuscript:

CBD	cannabidiol
ECS	endocannabinoid system
TRP	transient receptor potential
SODs	superoxide dismutases
CAT	catalases
GPx	glutathione peroxidases
Prx	peroxyredoxins
PUFAs	polyunsaturated fatty acids
MDA	malondialdehyde
4–HNE	4–hydroxynonenal
ANA	anandamide
TNF–α	tumor necrosis factor
LPS	lipopolysaccharides
IL	Interleukin
NHEK	normal human epidermal keratinocytes
NHDF	normal human dermal fibroblasts
HMOX1	hemooxygenase 1
PTX	paclitaxel
DOX	doxorubicin
DMS	dermal membrane structure
SNARE	Soluble NSF Attachment Receptor proteins
NSF	N–ethylmaleimide Sensitive Fusion protein
DOPE	dioleoylphosphatidylethanolamine
ROS	reactive oxygen species
Cys	cysteine
GSH	Glutathione
Nrf2	nuclear factor erythroid 2–related factor 2
Keap 1	Kelch–like ECH–associated protein 1
CAGR	Compound Annual Growth Rate
ECDD	Expert Committee on Drug Dependence
BHT	butylated hydroxytoluene
TRPV4	transient receptor potential cation channel subfamily V member 4
HU–331	Cannabidiol hydroxyquinone
FDA	Food and Drug Administration
EC	European Community
WHO	World Health Organization
PAMPA	Parallel Artificial Membrane Permeability Assay
5-HT1A	serotonin 1A receptor
